# Collagen as a double-edged sword in tumor progression

**DOI:** 10.1007/s13277-013-1511-7

**Published:** 2013-12-15

**Authors:** Min Fang, Jingping Yuan, Chunwei Peng, Yan Li

**Affiliations:** Department of Oncology, Zhongnan Hospital of Wuhan University, Hubei Key Laboratory of Tumor Biological Behaviors and Hubei Cancer Clinical Study Center, No. 169 Donghu Road, Wuchang District, Wuhan, 430071 China

**Keywords:** Collagen, ECM remodeling, Tension homeostasis, Traction force, Tumor progression

## Abstract

It has been recognized that cancer is not merely a disease of tumor cells, but a disease of imbalance, in which stromal cells and tumor microenvironment play crucial roles. Extracellular matrix (ECM) as the most abundant component in tumor microenvironment can regulate tumor cell behaviors and tissue tension homeostasis. Collagen constitutes the scaffold of tumor microenvironment and affects tumor microenvironment such that it regulates ECM remodeling by collagen degradation and re-deposition, and promotes tumor infiltration, angiogenesis, invasion and migration. While collagen was traditionally regarded as a passive barrier to resist tumor cells, it is now evident that collagen is also actively involved in promoting tumor progression. Collagen changes in tumor microenvironment release biomechanical signals, which are sensed by both tumor cells and stromal cells, trigger a cascade of biological events. In this work, we discuss how collagen can be a double-edged sword in tumor progression, both inhibiting and promoting tumor progression at different stages of cancer development.

## Introduction

Cancer is one of the most serious health threats worldwide, with an estimated 12.7 million new cases and 7.6 million cancer deaths each year [1]. Invasion and metastasis are the most fundamental properties of tumor biology and the root causes of cancer death. To tackle this problem, many efforts focusing on tumor cells have been made over the past century, and some genetic and epigenetic mechanisms have been elucidated [2–6]. Currently, with a general consensus on the significance of epigenetics, there has been a re-flowering of theory that cancer is a disease of imbalance, i.e., not merely a disease of rogue cells but the body's mismanagement of those rogue cells. It has been well documented that tumor microenvironment plays an important role in tumor progression via the co-evolution of tumor cells and tumor stroma [7–9]. Exploring the complex mechanisms of tumor progression from perspectives of tumor stroma has become a new frontier.

Of note is the extracellular matrix (ECM), a major component of tumor stroma, as a key regulator of cell and tissue function. Traditionally, ECM has been regarded primarily as a physical scaffold that binds cells and tissues together. However, recent studies have shown that ECM also elicits biochemical and biophysical signaling [10, 11] that affects cell adhesion and migration, tissue morphogenesis and repair, angiogenesis and cancer, and ECM proteolysis is tightly controlled in normal tissues but typically deregulated in cancer [8]. As the most abundant constituent of ECM, collagen accounts for the major function of ECM, and either increased [12] or decreased [13] deposition of collagen can be associated with increased malignancy.

This review summarized the dynamic interplay between collagen and tumor cells, focusing on changes in physico-chemico-biological properties of collagen. A new paradigm has been formulated that the intrinsic biomechanical forces in collagen can modulate ECM molecular conformation, producing either protective or destructive molecular and cellular events during tumor progression, depending on the stage of cancer development. Furthermore, the relationship between collagen and immune response and tumor angiogenesis is also explored.

## Basic structure and function of collagen

**Fig. 1:**
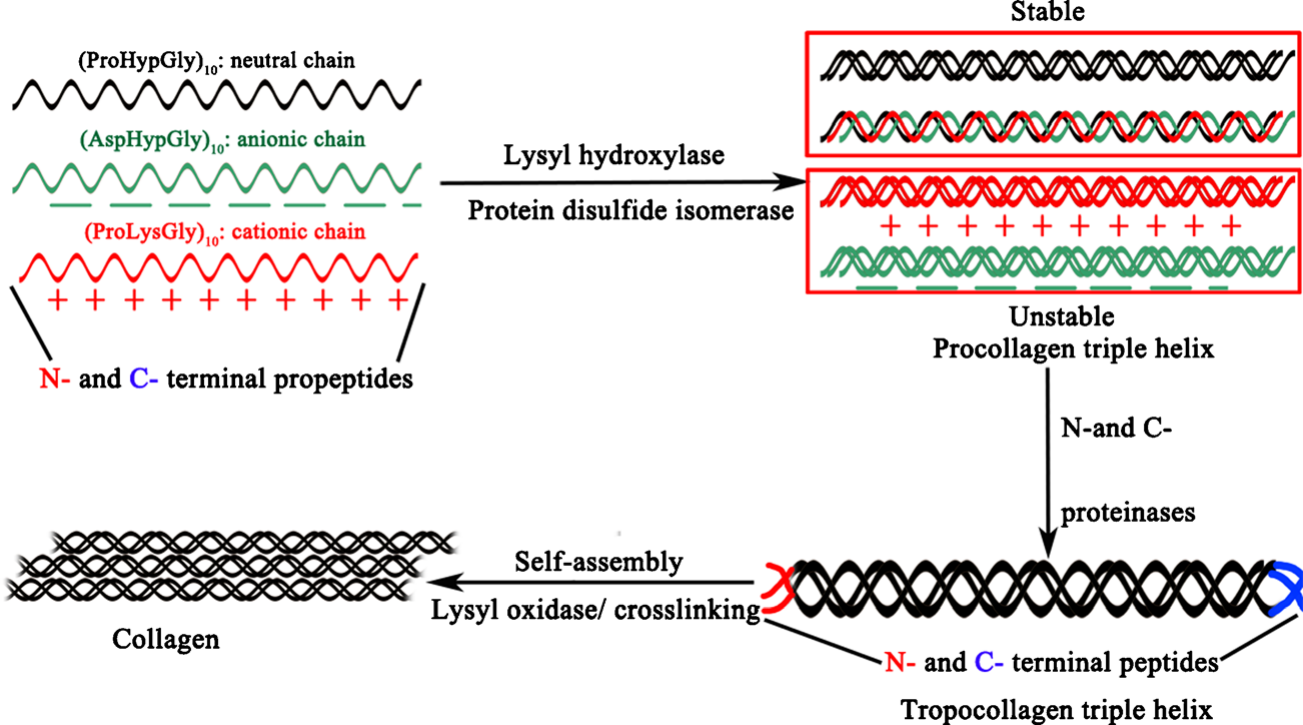
Biosynthesis of collagen. Three polypeptide α-chains each including an N- and C-terminal propeptides form triple helical structures called procollagen triple helix by lysly hydroxylase, protein disulfide isomerase and hydrogen bonds. Neutral strands are stable, but charged forms are unstable. Tropocollagen triple helix is formed as N- and C-terminal propeptides are converted into N- and C-terminal peptides by N- and C-proteinases. Under lysyl oxidase (*LOX*) cross-linking and self-assembly, collagen fibers or networks are formed

Collagen is abundant in humans accounting for one-third of total proteins. The fibrous, structural protein contains three polypeptide α-chains, displaying a polyproline-II conformation, a right-handed supercoil and a one-residue stagger between adjacent chains [14]. Each polypeptide chain has a repeating Gly–X–Y triplet, and the three polypeptide α-chains in the triple helix held together by inter-chain hydrogen bonds can be identical, but heterotrimeric triple helices are more prevalent than homotrimeric triple helices. Gauba and Hartgerink [15] observed that assembly of heterotrimeric triple helices was based on the 1:1:1 mixture of (ProLysGly)_10_/(AspHypGly)_10_/(ProHypGly)_10_ (Fig. 1). Collagen undergoes extensive posttranslational modifications by hydroxylation and cross-linking reactions in the endoplasmic reticulum prior to triple helix formation [16]. A number of enzymes and molecular chaperones assist in their correct folding and trimerisation, including hydroxylases, collagen glycosyltransferases, peptidyl *cis*–*trans* isomerase and protein disulphide isomerase [17, 18]. According to the structure properties of ECM, collagens can be categorized into classical fibrillar and network-forming collagen, FACITs (fibril-associated collagens with interrupted triple helices), MACITs (membrane-associated collagens with interrupted triple helices), and MULTIPLEXINs (multiple triple-helix domains and interruptions) [19]. At least 28 different types of collagens have been identified in vertebrates [19, 20] (Table 1). Among these, type I collagen is the archetypal collagen in that its triple helix has no imperfections and it has predominant role in tissue [16]. Others can have interruptions in the triple helix and do not necessarily assemble (in their own right) into fibrils. For example, MACIT has numerous interruptions in the triple helix, does not self-assemble into fibrils, and has roles in cell adhesion and signaling [20]. And Type IV collagen is the prototypical network-forming collagen. It forms an interlaced network at basement membrane (BM), found at the basal surface of epithelial and endothelial cells and essential for tissue polarity [21], where it has an important molecular filtration function.

**Table 1:** Collagens in vertebrates at a glance

Type	Class	Distribution
I	Fibril	Abundant and widespread in non-cartilaginous connective tissue: dermis, bone, tendon, ligament
II	Fibril	Cartilage, vitreous
III	Fibril	Co-distribution with collagen I: skin, blood vessels, intestine
IV	Network	BM
V	Fibril	Widespread and co-distribution with collagen I: bone, dermis, cornea, placenta
VI	Network	Widespread: muscle, bone, cartilage, cornea, dermis
VII	FACIT	Dermis, bladder
VIII	Network	Widespread: dermis, brain, heart, kidney
IX	FACIT	Co-distribution with collagen II: cartilage, cornea, vitreous
X	Network	Hypertrophic cartilage
XI	Fibril	Co-distribution with collagen II: cartilage, intervertebral disc
XII	FACIT	Co-distribution with collagen I: dermis, tendon
XIII	MACIT	Endothelial cells, dermis, eye, heart
XIV	FACIT	Widespread and co-distribution with collagen I: bone, dermis, cartilage
XV	MULTIPLEXIN	Located between collagen fibrils that are close to BM, capillaries, testis, kidney, heart
XVI	FACIT	Integrated into collagen fibrils and fibrillin-1 microfibrils, dermis, kidney
XVII	MACIT	Hemidesmosomes in epithelia
XVIII	MULTIPLEXIN	Associated with BM, liver
XIX	FACIT	Rare, localized to BM
XX	FACIT	Widespread: cornea (chick)
XXI	FACIT	Widespread: stomach, kidney
XXII	FACIT	Tissue junctions
XXIII	MACIT	Limited distribution: heart, retina
XXIV	Fibril	Shares sequence homology with the fibril-forming collagens: bone, cornea
XXV	MACIT	Brain, heart and testis
XXVI	FACIT	Testis and ovary
XXVII	Fibril	Shares sequence homology with the fibril-forming collagens: cartilage
XXVIII	Network	A component of the BM around Schwann cells, dermis, sciatic nerve

*BM* basement membrane, *FACIT* fibril-associated collagens with interrupted triple helices, *MACIT* membrane-associated collagens with interrupted triple helices, *MULTIPLEXINs* multiple triple-helix domains and interruptions

## ECM remodeling during cancer invasion

During cancer invasion, tumor stroma undergoes constant architectural changes, characterized by collagens degrading, re-depositing, cross-linking and stiffening in terms of ECM remodeling, and immune infiltration and re-differentiation of monocytes at the invasive front in terms of cellular changes.

### Increased deposition and cross-linking of collagens

The ECM scaffold undergoes considerable structural changes during tumor progression, including increased deposition of fibronectin, proteoglycans and collagens I, III and IV, and enhanced matrix cross-linking [22, 23]. Increased ECM deposition and remodeling creates a reorganized microenvironment to promote tumor progression by destabilizing cell polarity and cell–cell adhesion, and augmenting growth factor signaling [10, 24]. The progressive ECM remodeling produces typical morphological changes characterized by linearization of interstitial collagens at tumor invasion front, with significant impacts on tumor cell biology including gene expression, cell differentiation, proliferation, migration and responses to treatments [10].

**Fig. 2:**
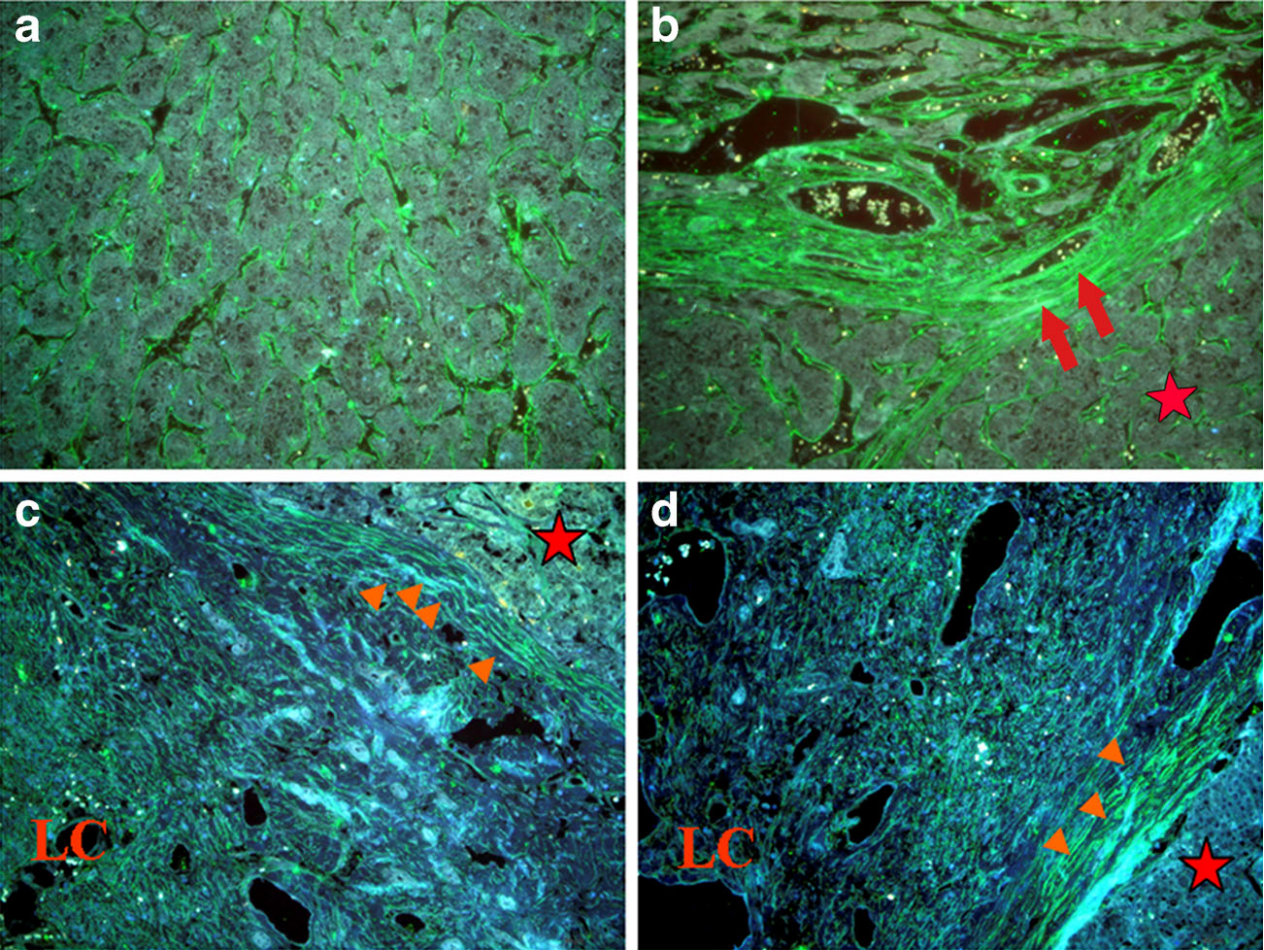
Type IV collagen expression demonstrated by quantum dot-525 (*green*). **a** Abundant type IV collagen fragments stochastically distributed in tumor tissues. **b** Rich type IV collagen in tumour stroma aligning with tumor nests. **c**, **d** Different characteristics between HCC (*red star*) and live cirrhosis (*LC*) tissues. *Red arrowheads* show stiff type IV collagen at interface of liver cirrhosis and tumor nests. *Red arrows* indicate the linear invasion "highways" for tumor cells escape. Scale bar = 50 μm

Breast cancer is a typical example of these changes. Clinicians have long recognized the connection between breast density and breast cancer risk [25]. Collagen surrounding normal epithelial structures in breast tissue is typically curly and smooth. However, parallel with tumor development, collagen progressively thickens, linearizes and stiffens which promotes metastasis by fostering cells migration into ECM. Indeed, intravital imaging shows that breast cancer cells and leukocytes migrate rapidly along collagen fibers [26]. Cancer cells might exploit these remodeled stiff collagens as invasion "highways", analogous to the preferential migration of glioma cells along the matrix associated with blood vessels and rigid myelin sheath bundles [27]. Our recent study also observed the linear invasion "highways" in hepatocelluar carcinoma (HCC) (Fig. 2).

#### Protease-dependent collagen cross-linking based on LOX

**Fig. 3:**
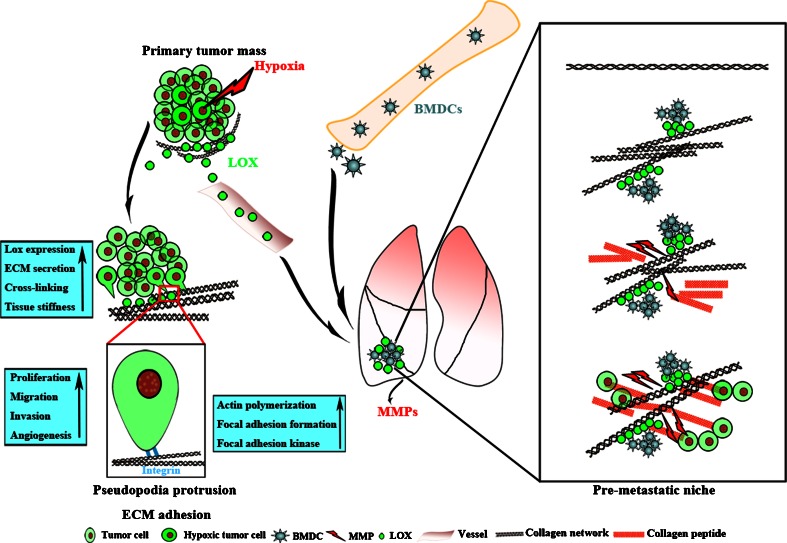
The role of LOX in tumor progression both in situ and distal organs. With tumor growth beyond 2 mm in diameter, prominent central hypoxia induces tumor cells to secrete LOX into tumor milieu. On the one hand, LOX-mediated type IV collagen cross-linking leads to ECM deposition and subsequent tissue stiffness, driving malignant progression predominantly by altering integrin focal adhesions and actomyosin- and cytoskeletal-dependent cell contractility. Tumor cells stretch pseudopodia protrusions with increased actin polymerization, focal adhesion formation and focal adhesion kinase that can in turn enhance tumor cells proliferation, migration, invasion, and perhaps tumor angiogenesis. On the other hand, LOX is disseminated into target organs (lung in this illustration) via circulation and deposits at terminal bronchioles and distal alveoli. The deposited LOX can crosslink type I and IV collagens to remodel ECM for recruiting BMDCs, so as to form the pre-metastatic niche

Protease dependent-ECM remodeling is predominantly catalyzed by enzymes such as lysyl oxidase (LOX) [28], synthesized by either stromal cells during early stages of carcinogenesis, or tumor cells during late stages of tumor progression in response to hypoxia [29]. As shown in Fig. 3, LOX, secreted by prominent central hypoxic tumor cells, can crosslink collagens and elastin, thereby increasing insoluble matrix deposition and tissue stiffness (Table 2). Increased ECM stiffness can activate integrins [30], enhance tumor cell adhesion and migration [31–33]. LOX is essential in driving tumor cells escape from primary site, extravasation and growth at secondary sites during metastasis [29, 34]. It is reported that LOX can be disseminated into distal target organs via circulation to mobilize bone marrow derived cells (BMDCs) to distal sites, and to create pre-metastatic niche [29], as evidenced by consistent correlation between increased LOX expression and higher cancer metastasis risk [34]. Furthermore, increased LOX expression is associated with early stromal reaction in breast cancer, and reactive fibrosis at the invasive front of infiltrating tumors also releases high levels of LOX [33]. The secreted LOX acts on collagen and increases integrin activity, stimulating tumor cells to stretch pseudopodia protrusions with increased actin polymerization, focal adhesion formation, resulting in the enhancement of actomyosin- and cytomyosin-dependent cell contractility and migration, leaving behind remodeled matrix tracks as the "metastasis highway" for tumor cells to travel. In more aggressive and poorly differentiated tumors, LOX also induces epithelial–mesenchymal transition (EMT) and promotes metastatic dissemination by facilitating tumor cells invasion into vascular system (intravasation) [35].

**Table 2:** Up-regulated expression of LOX in tumor tissues

Cancer type	Results	Function	References
Breast cancer	10-year DMSF^a^ low 10-year OS^b^ low	Activate HIF1-Akt pathway; mediate hypoxic control of metastasis; regulate actin filament formation; contribute to mechanotransduction-mediated regulation of TGF-β signaling; recruit BMDCs to form the pre-metastatic niche	[29, 34, 36]
Colorectal cancer	/	Correlated with absence of lymphovascular invasion; activate PI3K–Akt pathway to up-regulate HIF-1α protein synthesis	[37, 38]
Head and neck squamous cell carcinoma	5-year OS^c^ low	Strongly associated with increased metastasis, progression and death	[39]
Lung adenocarcinoma	5-year OS low	ECM remodeling; associated with advanced stage and metastasis	[40, 41]
Oral and oropharyngeal squamous cell carcinoma	10-year OS low	Independent prognostic biomarker and predictor of lymph node metastasis	[42]

^a^10-year DMSF: 10-year distant metastasis free survival

^b^10-year OS low: 10-year overall survival

^c^5-year OS low: 5-year overall survival

#### Protease-independent ECM stiffening

Non-enzymatic collagen cross-linking, such as glycation and transglutamination or increased biglycan and fibro-modulin proteoglycan deposition, can also stiffen matrix [36]. Such protease-independent ECM stiffening could be divided into several models. One kind is the excessive deposition of proteoglycans. It could contribute to fibrosis by parenchyma stiffening in injured lungs [37], and be accompanied with elevated risk of developing cancers in diabetic patients with inappropriate glycation-mediated cross-linking [38–40]. Another process is fibronectin-mediated collagen reorganization [48]. The size, density and rigidity of fibronectin in vivo influence function of collagen, and dynamic and reciprocal interactions between collagen and fibronectin likely induce tumor progression [41]. Indeed, fibronectin has been implicated as early step of cancer metastasis [42]. And secreted protein acidic and rich in cystine (SPARC), a highly conserved, multi-functional glycoprotein, produced both by cancer cells and stromal cells, can also be involved with such protease-independent ECM stiffening models. It could participate in ECM organization and bind to type I and IV collagen [13] and also suppress or promote progression of cancers depending on interactions at cell-matrix and tumor-stroma surface [13, 43–46].

### Increased degradation of collagen

Collagen in tissues has been traditionally regarded as merely a physical barrier against cancer invasion and tumor cells migration [47, 48]. The prerequisite for tumor invasion is collagen degradation [49], for which matrix metalloproteinases (MMPs) play an important role [8, 50], with direct causative effects on tumor growth, invasion and angiogenesis [51–63], by a host of mechanobiological mechanisms such as to degrade collagen paving a potential tunnel for escaping tumor cells [8], to disturb tumor microenvironment producing additional mechanical force to induce EMT, and to expose active sites on collagen to recruit monocytes leading to a cascade of innate immuno-inflammatory reactions [54]. However, there is diversity of tumor invasion mechanisms, in which collagen degradation plays different roles. In single cell/amoeboid migration, cells tend to migrate in the absence of proteolytic ECM breakdown by adapting their shape to and squeezing through tissue gaps and trails. In mesenchymal migration, invading cells adopt spindle-shaped, elongated morphology with focalized cell-matrix adhesions containing multi-molecular integrin clusters and proteolytic activity toward ECM substrates. Focalized proteases on the cell's surface generate small microtracks through which subsequent cells can follow. In collective invasion, one or several leader cells with mesenchymal characteristics, such as fibroblasts, form the tip of multi-cellular strands and generate forward traction and pericellular proteolysis toward the tissue structure [49].

Recent studies have gained new insights into the function of MMPs with a new paradigm for mechanobiological mechanisms in tumor invasion [55], which will be described in detail below.

### Reversible changes of BM: opening the door for non-proteolytic ECM migration

At initial phase of tumor invasion, BM is breached as tumor cells invade into interstitial tissue and colonize distant organs. Although proteolysis-dependent collagen degradation is important for this process, there is much evidence showing that proteolysis is dispensable in the BM transmigration events [56, 57].

In this process, the putative mechanism underlying reversible BM remodeling could rely on a precedent wherein endothelial cells function as the gatekeeper of transmigration by flexibility on BM [58]. That is, endothelial cells can generate traction force in response to signals during tumor cell–endothelial cell adhesive interactions, which regulate collagen structure and organization. Indeed, BM has been proposed to display thixotropic properties: an increasing force generates a change in BM viscosity, altering BM permeability to macromolecules and perhaps even cells [59]. So, in such a scenario, reversible disruptions of type IV collagen quaternary interactions are required for "closing and opening" BM, permitting non-proteolytic transmigration to occur without enzymatic degradation [60]. In this manner, a reversible system might be envisioned wherein tumor cells or others such as monocytes can be accommodated to pass through. Thus, collagen can regulate immune cell infiltration into tumors. Hence, cooperation between traction forces and the activity of cell surface enzymes on either the endothelial cell or tumor cells themselves theoretically would enable the reversible opening and closing of BM [61, 62].

Thus, collagen increase and decrease are both involved in tumor progression, and these two processes are coordinated reciprocally to promote tumor invasion and metastasis. First, LOX mediated collagen cross-linking can recruit some stromal cells to adhere and secrete more MMPs. Then, MMPs can degrade collagen to expose active sites to generating a pro-tumorigenic microenvironment to facilitate tumor progression. Although direct mechanisms have not been elucidated, MMPs are associated with LOX expression in breast cancer [29]. LOX mediated collagen cross-linking seems to function in synergy with MMPs, which may lead to ECM remodeling favoring tumor progression.

## Tension homeostasis and tumor progression

Force is essential for normal tissue-specific development, in which it regulates cell survival, growth and migration, and orchestrates tissue organization and function. Increased matrix cross-linking and ECM protein deposition or parallel reorientation of matrix collagen can stiffen tissue locally to alter surrounding cells growth or drive cell migration. Loss of tissue homeostasis and mechanoreciprocity is a hallmark of disease. Although much is known about biochemical pathways that direct cell behavior, by comparison, little is known about how force regulates cell fate and tissue phenotype. Two primary cellular mechanisms involved in tumor invasion and migration are cellular physical rearrangement and reorientation of collagen by traction forces generated by epithelial cells [63, 64], the consequence of EMT and cellular catabolism of ECM by enzymatic cleavage of collagen [65–67]. Both can be regulated by tissue tension. Here we take a multiscale approach to describe tension homeostasis changes present at tumor–stroma interface, ranging from the molecular level (collagen and specific enzymes secreted by tumor and stromal cells involved in collagen reorganization) and the cellular level (tumor and stromal cells) to the structural level (ECM) [65, 68, 69].

### EMT process

**Fig. 4:**
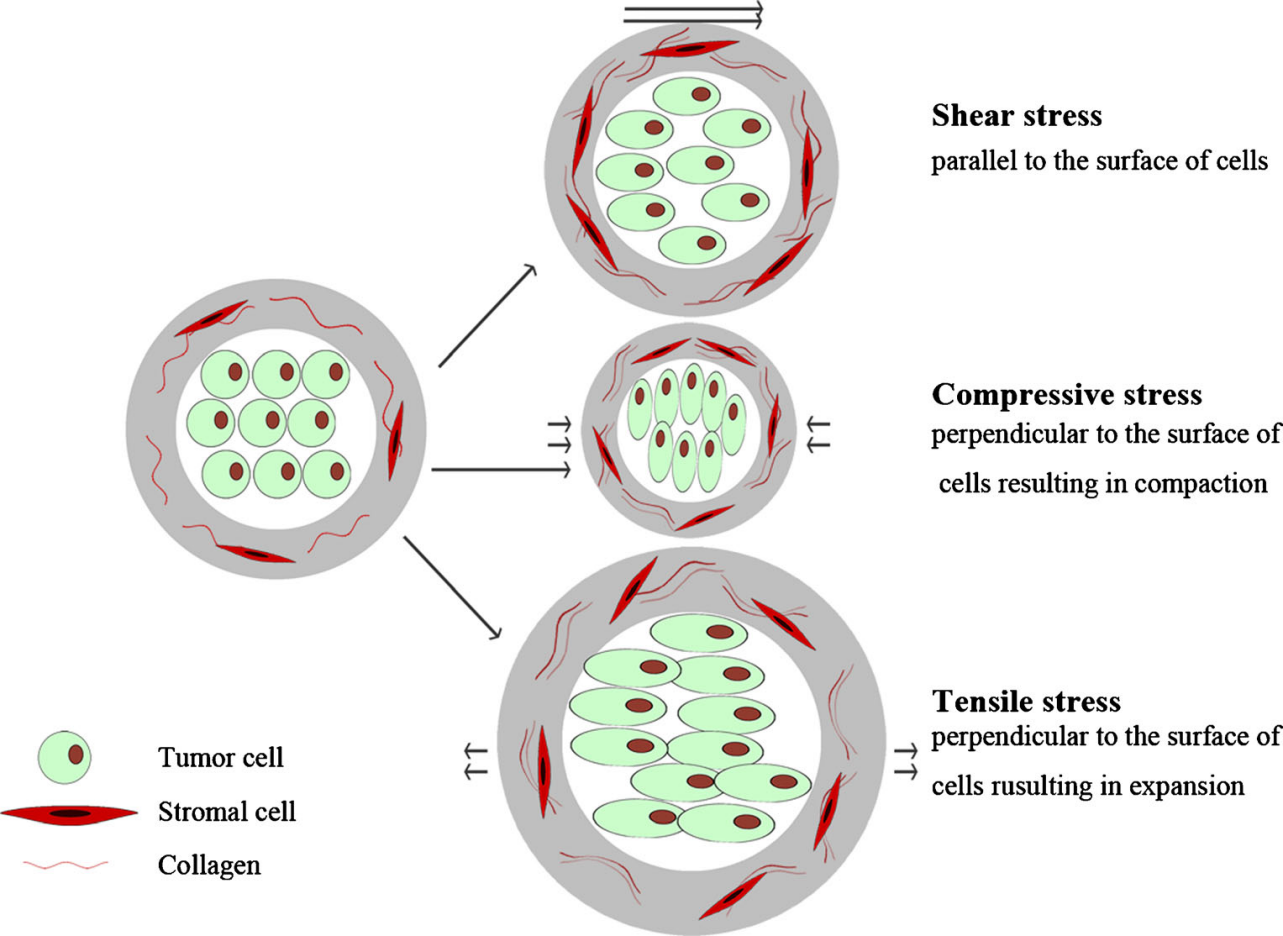
Force applied to deform and influence the biological behavior of tumor cells. Tissue microenvironment can exert three forms of force on tumor cells, including shear stress, compressive stress and tensile stress

Interactions between tumor cells and their surrounding ECM are recognized as primary forces driving the EMT process [68]. Imbalanced biomechanical force at tumor–stroma interface is the key trigger initiating EMT, and ultimately leads to tumor cells escaping [55]. Tumor cells dynamically adapt to the force (Fig. 4) by changing their behaviors and remodeling their surrounding microenvironment. As the tumor mass expands at early stages of the invasion, collagen in stroma will realign and stretch perpendicular to the mass to resist tumor expansion and enzymatic degradation. Thus, tumor cells must overcome increased collagen alignment and density before invasion and migration. With the tumor mass expanding, stress on ECM increases correspondingly, until reaching a critical point, termed as the biomechanical trigger [62], which can be sensed by both tumor and stromal cells through mechanoreceptors. In turn these cells exert actomyocin- and cytoskeletal-dependent traction forces on ECM [70–72]. Eventually, tumor and stromal cells deform as consequences to the altered tissue tension [73, 74], the expanding tumor mass [24], matrix stiffening [10], and increased interstitial pressure due to a leaky vasculature and poor lymphatic drainage, initiating EMT [75]. These deformed cells acquire a more spindle-like fibroblastic morphology, less adhesive properties, enhanced motility and invasive behavior.

### Tension as a regulator for MMPs function

**Fig. 5:**
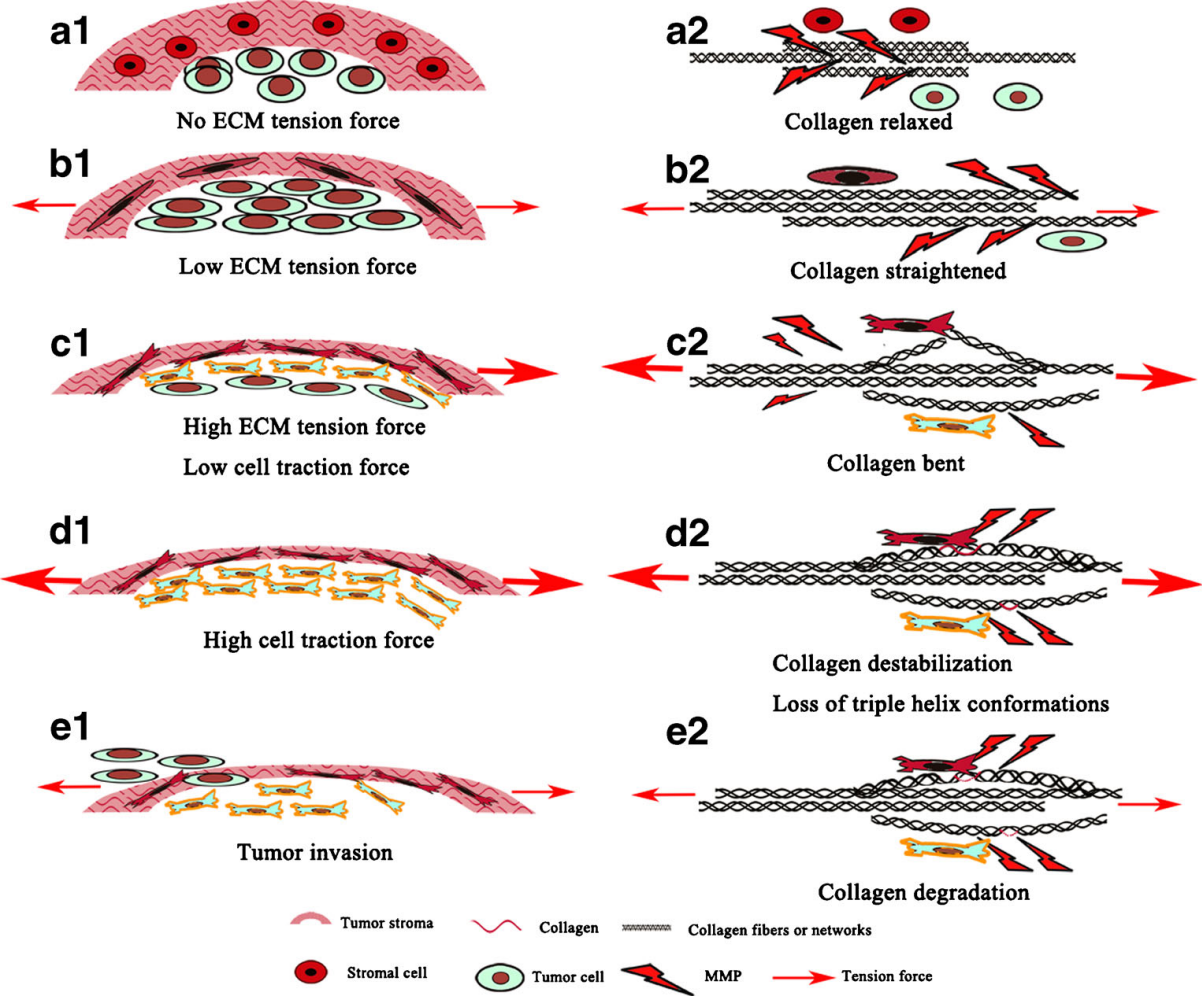
A paradigm for how tumor and stromal cells interact to degrade ECM and change tensions for tumor invasion. **a1** Dormant tumor cells without tension force; **a2** collagen relaxed and elastic. **b1** With tumor growth, low tension force exerts on collagen which stretches accordingly; **b2** Entrance hole for MMPs-dependent cleavage is closed as collagen stretches. **c1** As the tumor continues to expand, increasing tension force transmits signals to both tumor and stromal cells to remodel ECM in order to reduce tension force. Tumor and stromal cells undergo EMT process which in turn increase their traction force; **c2** collagen bends and changes conformational structures correspondingly. **d1** High traction force exerted by cells destabilize the stroma; **d2** tumor and stromal cells attach to collagen and unwind triple helix, exposing sites for cleavage by MMPs. **e1** Tumor invasion and metastasis occur with degradation of collagen; **e2** MMPs enter into triple helix to cleave α-chains

ECM degradation is necessary for tumor invasion. However, collagen as one of the most abundant components of ECM is the largest, strongest and most difficult to penetrate by tumor cells due to limited degradation by only a few MMPs. And also collagen undergoes remodeling and reorientation in response to tumor mass expansion, with a concomitant increase in collagen density and mechanical tension to constrain tumor expansion [70, 76]. Re-constructed collagen can prevent them from being degraded by MMPs. As shown in Fig. 5, in normal tissues, MMPs can access into collagen to degrade (Fig. 5a1–a2). However, in tumor tissues, function of MMPs is regulated by the tumor microenvironment, here accenting the coordination of stromal cells, tumor cells and collagen as well: (1) initially, increased tension in ECM is protective, it makes the collagen stretched, in that it inhibits MMP-related collagen cleavage as collagen undergoes molecular conformation changes, such that the enzymes no longer have access to the cleavage site on collagen as the binding sites hidden (Fig. 5b1–b2); (2) with the tumor extensively expansion, tumor cells and stromal cells sense high tension in ECM so as to exert traction force on collagens to make them deform through integrin binding (Fig. 5c1–c2); (3) as tension exerting on cells increases with further tumor mass expansion until a critical point, rather than being protective for ECM degradation, increasing tension becomes a key biomechanical trigger for tumor and stroma cells to remodel the ECM. Via surface integrin receptors, cancer cells can sense mechanical signals of increasing tension in the microenvironment, and respond by increasing their traction forces on ECM, resulting in collagen triple helix separation (Fig. 5d1–d2); (4) thus, entrance hole for MMPs is opened by destabilizing collagen triple helix and unwinding collagen molecule (single α-chain) [62], accompanied with tension decrease in ECM (Fig. 5e1–e2).

Thus, during tumor progression, ECM remodeling contributing to tension changes is important for both triggering EMT and regulating MMPs. As the tumor expands, the tension in ECM will increase correspondingly, until reaching a critical point [55]. This may be a turning point for most tumors and before reaching this turning point, tumor progresses slowly, but beyond this turning point, tumor progression is accelerated.

## Collagen as a regulator for tumor associated immune infiltration

Collagen is not just a passive player during tumor progression. Recent experimental developments point to a far more complex role for these structure proteins. A variety of immune cells are present in cancers and many of these accumulate and migrate within regions of dense collagen [26, 77, 78].

**Fig. 6:**
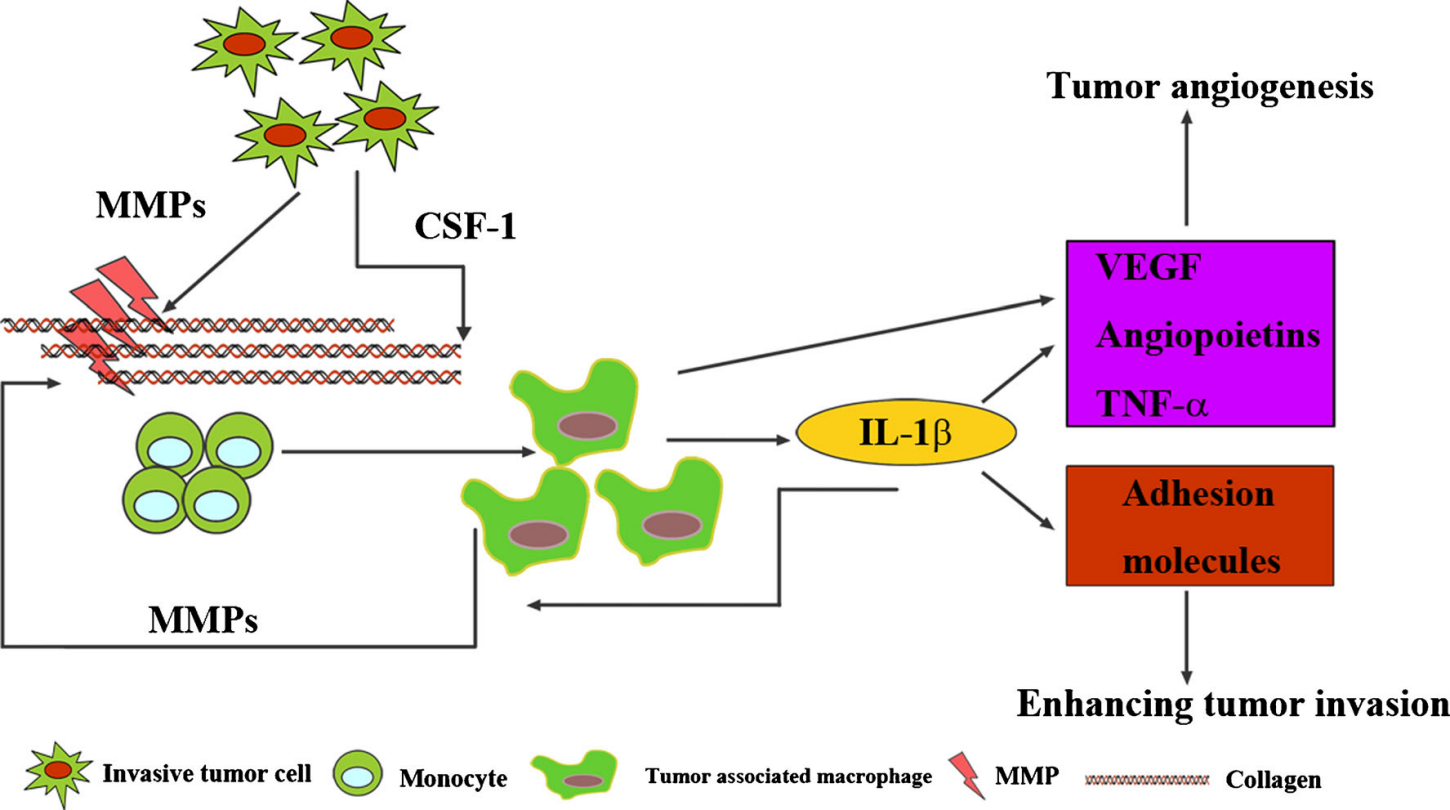
Collagen regulates tumor associated immune infiltration. MMP-dependent collagen fragments can recruit monocytes and further promote them to differentiate into TAMs with the help of CSF-1. TAMs themselves secret factors responsible for tumor progression, including tumor angiogenesis. Meanwhile, they themselves can activate MMPs to degrade collagens

For example, macrophages in and around tumor nests can, in principle, either promote or inhibit tumor progression [79], and collagen plays a crucial role in regulating the balance between the tumor-inhibiting and promoting effects of macrophages. It is reported that culturing macrophages on type I collagen reduces their cytotoxicity against tumor cells [80], suggesting that collagen inhibits the differentiation of the macrophages to the tumoricidal M1-like type. The possibility that collagen scaffolds can regulate macrophages polarization is further supported by the increase in pro-tumorigenic, M2-like macrophages observed in tumors of Sparc^−/−^ mice with abnormal collagen scaffolds [13]. The principal factors behind this transformation appear to be interleukin-6 (IL-6) and colony-stimulating factor 1 (CSF-1) [81]. And it is also known that collagen degradation products serve as chemotactic stimuli for monocytes [82, 83]. As collagen is degraded during metastasis, the resulting collagen fragments may recruit tumor associated macrophages (TAMs). These TAMs are abundant in most solid tumors [84], predicting poor prognosis [85]. As shown in Fig. 6, monocytes are recruited into collagens degradation areas accompanied with MMPs release by tumor cells [85]. After releasing soluble CSF-1, monocytes differentiate into TAMs promoting tumor growth and metastasis [85]. Similarly, IL-6 will inhibit monocytes differentiation into dendritic cells, directing immune responses against tumor cells [85]. TAMs themselves express factors in response to tumor progression, promoting tumor angiogenesis, invasion and intra-and extravasation [86, 87]. For example, IL-1β, a special form of IL-1, can stimulate expression of VEGF and TNFα to promote tumor angiogenesis and adhesion molecules, including intercellular-adhesion molecule 1 (ICAM-1), vascular cell adhesion molecule 1 (VCAM-1) and E-selectin, to enhance invasion. And IL-1β can also activate MMPs to degrade collagen [88, 89]. Thus, all these have uncovered a significant positive feedback in immune responses to cancer-related collagen degradation and indicate the link between degraded type IV collagen and tumor progression [90].

In addition, ECM stiffness could influence T cell activation via integrin-mediated adhesions assembly promotion [10, 90] and collagen-mediated activation of leukocyte-associated Ig-like receptors (LAIRs). LAIRs are highly expressed on most immune cells and can through their immunoreceptor tyrosine-based inhibition motifs (ITIMs) inhibit immune cell activation [91]. Although it is not clear whether LAIRs and integrins cooperate, activation of LAIRs is a plausible mechanism whereby high levels of deposited collagen lead to inhibition of an anti-tumor immune response.

As collagen influences immune cell infiltration, immune cells also influence collagen architecture. Macrophages regulate mammary epithelial invasion during tissue development [92]. This may in part be achieved through their ability to initiate the remodeling and reorganization of collagen surrounding the developing epithelium [93], and secretion of a repertoire of soluble factors such as MMPs. Macrophages can also take up collagen for intracellular degradation via binding to the glycoprotein [94, 95].

## Collagen and tumor angiogenesis

Angiogenesis, a specialized form of branching morphogenesis wherein endothelial cells detach themselves from the existing vasculature, invade surrounding tissues, and reorganize into patent tubules [86, 96], is vital for tumor growth and metastasis. Tumor angiogenesis is characterized by the secretion of multiple pro-angiogenic factors to trigger the angiogenic switch resulting in the development of a structurally and functionally abnormal vasculature.

Collagens are essential for tumor angiogenesis. Inhibition of collagen metabolism has been demonstrated to have anti-angiogenic effects [97], confirming that blood vessel formation and survival are indispensably connected with proper collagen synthesis and deposition at BM [97]. Interactions between endothelial cells and ECM, in particular collagen IV in the vascular basement membrane (VBM) play key roles in regulating angiogenesis [21]. For instance, type IV collagen could modulate (promote/inhibit) endothelial cells growth and proliferation [98]. The in vitro endothelial cells culture experiments have shown that triple-helical fragments of type IV collagen could stimulate endothelial-cell adhesion and migration as active as intact type IV collagen, while the noncollagenous domain 1 (NC1 domain) of type IV collagen alone is insufficient to mediate endothelial-cell migration [99, 100]. And a further research of NC1 domains indicated that they are typical anti-angiogenic molecules, which could inhibit endothelial cells migration, proliferation and tube formation by competing with intact type IV collagen for binding integrin [101]. In addition, studies on in situ carcinoma demonstrated that MMP-mediated degradation of BM could expose cryptic domains of type IV collagen with pro-angiogenic activity, in the early stage of local tumor progression [102, 103], and generate type IV collagen fragments with anti-angiogenic activity, such as arrestin, canstatin and tumstatin in the late stage [104–106]. Thus the structural integrity of collagen IV is of utmost importance for tumor angiogenesis [21].

## Conclusions and future perspectives

Over the last 10 years, cancer research has been increasingly shifted to the tumor microenvironment. In particular, ECM, the intermediary between biomechanics and tumor biology, can mediate dual roles as tumor suppressors at the early stages but paradoxically as tumor promoters at the later stages of tumor progression. Current researches of ECM focus on biochemical mechanisms associated with tumor progression, namely the intracellular pathways of signal transduction from the ECM to the nucleus (outside-in signaling), and the cellular metabolic responses for synthesizing proteinases to degrade (inside-out signaling) [62]. However, little attention has been paid to the dynamic changes of ECM biomechanics accompanying these biochemical events. With the increasing appreciation that biomechanical forces are crucial determinants for tissue development, cell differentiation and homeostasis, it is reasonable to conclude that loss of the ability to sense, respond and adapt appropriately to such biomechanical forces, on the part of tumor cells and stromal cells, contributes to tumor progression. Therefore, collagen as the most important architecture of ECM to generate these biomechanical forces, is no longer considered as a static and passive background upon which metastasis takes place. To elucidate how the changes in collagen structure and the related biomechanical forces to modulate tumor invasion and metastasis, thus deciphering the "collagen code" in cancer progression, is an intriguing field for intensive investigation.

## Abbreviations

ECM: Extracellular matrix
FACITs: Fibril-associated collagens with interrupted triple helices
MACITs: Membrane-associated collagens with interrupted triple helices
MULTIPLEXINs: Multiple triple-helix domains and interruptions
BM: Basement membrane
HCC: Hepatocelluar carcinoma
LOX: Lysyl oxidase
BMDCs: Bone marrow derived cells
EMT: Epithelial–mesenchymal transition
SPARC: Secreted protein acidic and rich in cystine
MMPs: Matrix metalloproteinases
IL-6: Interleukin-6
CSF-1: Colony-stimulating factor-1
TAMs: Tumor associated macrophages
LAIRs: Leukocyte-associated Ig-like receptors
ITIMs: Immunoreceptor tyrosine-based inhibition motifs
VBM: Vascular basement membrane
NC1 domain: Noncollagenous domain 1
